# Lessons Learned from the COVID-19 Outbreak as Experienced by ICU Nurses: Manifest Qualitative Content Analysis

**DOI:** 10.3390/healthcare11091269

**Published:** 2023-04-28

**Authors:** Hind Alharthi, Hanan A. M. Youssef, Abdulellah Al Thobaity, Ruba W. Yassen, Ebaa M. Felemban, Mohammed Almalki, Modi Al-Moteri

**Affiliations:** Nursing Department, College of Applied Medical Sciences, Taif University, P.O. Box 11099, Taif 21944, Saudi Arabia

**Keywords:** ICU, nurses, pandemic, COVID-19, family

## Abstract

From the moment the World Health Organization (WHO) declared the coronavirus (COVID-19) a global pandemic, intensive care unit (ICU) nurses struggled to care for the COVID-19 patients. As the pandemic became increasingly critical, the task of daily care for critically ill patients fell upon the shoulders of ICU nurses. Understanding the lived experience of ICU nurses, as well as their perception of the experience, is important to identify key lessons to further improve ICU nurses’ psychological well-being and resilience. This study explored the lived experiences of ICU nurses who cared for COVID-19 patients using exploratory manifest qualitative content analysis. The findings of this study revealed that these ICU nurses offer important information concerning the crucial role of nurses’ family support and religious–spiritual practices in helping them to maintain well-being and cope with the intense situations caused by the pandemic. The lessons emerging from the study findings show that family support and religious–spiritual practices are resources for coping and resilience in times of future pandemics. This implies that ICU nurses who provide care during stressful emergency surges require relief by nurses working in other areas to provide them the opportunity to rest, reconnect with family and become emotionally re-energized.

## 1. Introduction

Since the coronavirus pandemic spread around the globe, the rate of intensive care unit (ICU) admission of patients has increased significantly [1]. Nurses caring for COVID-19 patients have been directly exposed to extreme anxiety and tensions, and they have described the working environment as emotionally draining [2]. ICU nurses have treated great numbers of critically ill COVID-19 patients, providing end-of-life comfort for patients without their families in attendance and battling almost persistent stress, fears and issues with ethical dilemmas [3]. They have provided care for infected patients despite their tiredness, fear of becoming infected or transmitting the infection to their family members, illness of their colleagues and the loss of their patients. This situation can pose the risk of the development of psychological problems [2]. The literature has shown that ICU nurses can experience stress signs such as sleep disturbance, depression and anxiety [4]. 

The negative impacts of COVID-19 on ICU nurses’ well-being have been well documented [5,6,7]. Indeed, there are numerous published studies that qualitatively investigated nurses’ experience during the pandemic [8,9,10,11]. However, most of these studies were based on interpretive approaches in which the findings were analyzed through the researcher’s values point of view and interpretation, and hence say nothing about the nurses’ own experience [12]. Realistic conclusions from these studies might be lacking from policy changes including devising educational, supportive, protective and compensation programs for ICU nurses affected by the pandemic. It is of interest and significance to understand how ICU nurses experienced the pandemic. Therefore, ICU nurses were chosen in the present study as study subjects, and the exploratory manifest qualitative content analysis was applied to understand the lived experience of ICU nurses, as well as their perception of the experience, to identify key lessons to further improve ICU nurses’ psychological well-being and resilience.

### Study Aim

This study explored the lived experiences of ICU nurses who cared for COVID-19 patients using exploratory manifest qualitative content analysis.

## 2. Materials and Methods

This study employed an exploratory manifest qualitative content analysis. A manifest content analysis is a qualitative approach to explore the occurrence and repetition of certain phrases or words with the purpose of understanding the phenomena, yet not interpreting the meaning [13]. This approach provides direct answers for research questions through exploring the lived experience of individuals in everyday language, with words serving as an analytical tool, not an interpretive structure [14]. It seems appropriate to use this approach to explore the commonality of the responses among ICU nurses when reporting their experiences during the COVID-19 pandemic. 

### 2.1. Data Source

This is a secondary analysis of existing data, obtained from a larger quantitative study conducted after the end of the 1st wave and during the 2nd wave of COVID-19 to screen the presence of posttraumatic stress disorder (PTSD) symptoms among ICU nurses who were assigned in COVID-19 hospitals.

The original quantitative study was conducted by the principal researcher of the current study as a part of her master thesis project. According to Cheng and Phillips [15], any additional analyses of data collected for specific research purposes are considered “secondary analyses of existing data”. Cheng and Phillips [15] claimed that this is an important approach to maximize the output of the collected data via assessing more variables than those precisely identified to answer their original research questions.

The existing data included (*n* = 114) ICU nurses who participated in an unidentified online survey conducted in English, to screen the presence of PTSD symptom using the civilian version of the 17-item PTSD Checklist (PCL-C) [16]. The 17-item checklist covers three main syndromes of intrusion, avoidance and hyperarousal, on a 5-point Likert scale (1 = Not at all; 2 = A little bit; 3 = Moderately; 4 = Quite a bit and 5 = Extreme).

All ICU nurses who participated in the online survey were given the chance to freely write an open-ended short response about their COVID-19-related experience. Eighteen participants completed the open-ended questions and responded to the following:

- Describe your experience after having worked in COVID-19 settings.
- Describe the impact of COVID-19 on your psychological health.
- Describe strategies you used to cope with the experience during that period.

These open-ended questions were constructed on the basis of previous research conducted at the beginning of the pandemic [17,18,19] and aimed to explore in greater depth the concept of posttraumatic stress disorder. However, due to the time restraint, these data were not used or analyzed by the larger quantitative study researcher team. Hence, the current study utilized the textual information as a data source for the current analysis. ICU nurses who participated in the online survey consented to reuse their online survey response anonymously for future research and non-commercial purposes. The overall results of the larger pool of data were used elsewhere for other research purposes.

### 2.2. Study Setting and Context

During the COVID-19 pandemic, the Kingdom of Saudi Arabia Ministry of Health (MOH) declared some of its hospitals to be completely “COVID-19” hospitals. In the western region, hospital ‘A’ and hospital ‘B’ were identified as COVID-19 hospitals. The larger data gathering took place at these two hospitals. Hospital ‘A’ is a 500-bed facility with 30 ICU beds, while hospital ‘B’ is an 800-bed facility with 27 ICU beds. There are three shifts for the nurses in both hospitals, morning (7 a.m.–3 p.m.), evening (3 p.m.–11 p.m.) and night (11 p.m.–7 a.m.). All nurses (*n* = 180) working in an adult ICU at these two regional governmental hospitals were eligible and invited to participate in this study.

### 2.3. Data Collection

The process of data collection for the larger pool of data started by sending an invitation email message with a URL-embedded online survey and recruitment materials. Recruitment materials included a statement describing the purpose of the study, an ethical approval statement and the principal researcher’s contact information. Potential participants were instructed that they are free to participate and free to withdraw at any point by sending a withdrawal email request to the principal researcher by email. Once they click the link, they are required to respond to an unidentified online survey.

### 2.4. Analysis

Manifest content analysis was utilized. The textual information obtained from the open-ended short response was less condensed and to some degree abstracted by the individuals who wrote of their experience. According to Lindgren et al. [13], if the qualitative study aims to explore individuals’ experiences of a phenomena that is close to the individuals’ lived experience, it would be appropriate to limit the analysis to categories at a descriptive level. A category describes the content on a visible level, with a low degree of interpretation and abstraction [20]. The content analysis of the study starts by reading and rereading of the responses to be familiar with the data. Similar words were counted to understand their occurrence [21]. Then, similar words were grouped to develop codes (Table 1). Finally, similar codes were grouped into categories that reflect a shared meaning [21,22]. 

### 2.5. Data Rigor

Rigor of the current study findings was ensured through maintaining the credibility, transferability, dependability and confirmability [23]. Credibility was confirmed by two of the study researchers who independently and manually counted the phrases and completed the coding and categorization process without using software assistance. However, the two researchers met frequently to discuss and come to an agreement upon initial codes and categories. Findings were then rechecked by two other researchers for confirmation. Transferability was ensured through selecting quotes that best described the category [24]. Meanwhile, confirmability was maintained by reviewing the overall codes, categories and quotations in relation to data set. Finally, dependability was maintained by making sure that researchers included all the analysis steps with sufficient description to ensure the work can be repeated by another researchers. 

## 3. Results

Of the 18 ICU nurses who completed the open-ended questions, 89% (*n* = 16) were female of which 69% (*n* = 11) were married. The remaining 11% (*n* = 2) were unmarried male participants. As this is a secondary analysis of existing data, no statistical relationship was sought between the findings and the demographic data. However, Cheng and Phillips [15] asserted that to conduct a secondary analysis of existing data, researchers must obtain a detailed description of the population under investigation. Hence, 114 ICU nurses participated in the original quantitative study. Of which, the age of 86% (*n* = 98) ranged from 20 to 40 years, while the remaining (14%) were above 40 years old. More than half of the sample 52% (*n* = 59) had five (5) to less than five years of work experience in ICU. The majority of participants (97%) were women (*n* = 110), and 54.3% (*n* = 62) were married. Almost 24.5% (*n* = 28) of participants worked more than 40 h per week during the peak of the COVID-19 pandemic first wave. The number of participants holding a bachelor’s degree in nursing was higher than those holding an associate degree in nursing, (82.5% (*n* = 94) and 14.8% (*n* = 17), respectively). Around 97% (*n* = 111) of participants had provided direct care to a COVID-19 patient. In addition, approximately 54% (*n* = 61) were considering leaving their job, whereas 4% (*n* = 4) seriously thought about leaving work. PCL-C screener responses were categorized to identify individual meeting symptom criteria for PTSD using the procedure recommended by Palmieri and colleagues [25]. Mainly, participant symptoms receiving a score of 3 or greater were considered as having symptom presence. Following this scoring strategy, 47 (41%) of ICU nurses met screening criteria after the end of the 1st wave and during the 2nd wave of COVID-19.

### 3.1. Findings

Manifest content analysis of the open-ended short responses identified 30 different word(s)/phrase(s). The frequency of the appearance of some word(s)/phrase(s) in the text reached up to 16 time(s), reflecting commonality of the responses. For instance, “speak to family member to relieve stress” appears 16 times; “reading/ listening to holy book(s)” appears 13 times; “it was hard staying away from kids” appears 10 times; and “caring for more critical patients” appears 10 times. The high number of repetitions may be indicative of importance as shown in Table 1.

These words were grouped to form 16 basic codes which could be grouped into four (4) categories: (1) family-related stressful experience, (2) work-related stressful experience, (3) stress-related psychosocial signs and (4) stress-related coping strategies.

#### 3.1.1. Family-Related Stress Factors

The first category was family-related stressful factors which consisted of four codes: (1) family separation, (2) fear of transmitting infection to vulnerable family members, (3) death of a loved one and (4) marital problems. In the pandemic processes, the consequences of the crisis on nurses and their relationship with their families were obvious in the study findings. Indeed, the ICU nurses who participated in the study increasingly reported experiencing a stressful time due to the COVID-19 pandemic. Participants classified themselves as high risk for getting infected with COVID-19. They reported that they imposed self-isolation and lived apart from their family for the sake of protecting them.

Some nurses reported that they “… left their kids with parents …”, “… could not approach, see or be near their mothers, …. fathers, … sisters, … brother, …” and experienced “… being apart from husband …”. Some nurses described such separation as “… the hardest part of the pandemic. ICU nurses reported that the only concern they kept on thinking about was “… bringing infection to their beloved one …” and reported that they were “…. terrified …”. The impact that the COVID-19 pandemic had on ICU nurses’ personal life was often intense. One nurse “… lost her mother…”, and she reported that her mother’s death has created “… a permanent wound …” in her “… heart”. One nurse reported that after isolation ended, she found herself “… constantly fighting …” with her “… husband”.

#### 3.1.2. Work-Related Stress Factors

The second category was work-related stressful experiences and consisted of three codes: (1) workload, (2) patient(s) death and (3) death of a colleague(s). Most of the ICU nurses who participated in the study reported increased workloads during the pandemic. Some nurses reported “… long work hours with no time for rest …”, “… less of days off …”, “… handling more critical patients …” and “… frequently dealing with sudden worsening of patients’ condition …”. One nurse reported “… feeling sadness …” due to the “… death of the best physician they have in the unit whom she works with …”. Another nurse reported that “… number of deaths has increased …” and that “… the death of young patients is not something that he used to deal with …”.

#### 3.1.3. Stress-Related Psychosocial Signs

The third category was stress-related psychosocial signs and consisted of five codes: (1) sleeping difficulties, (2) distress, (3) social isolation, (4) fear and (5) helplessness. In pandemics, ICU nurses have higher levels of distress compared to the entire society. This was clear in the current study as most of participants’ statements indicated that they were under certain degree of psychological stress. Participants described the pandemic experience as a “hard time”. They expressed a lack of interest in interactions with others. For instance, two nurses reported they “… avoid joining family, friends’ occasions …”. Some participating nurses reported “… difficulty in falling in sleep …”. One nurse reported that she “…. would prefer to be alone rather than with other people …”. One ICU nurse has a feeling of “… constant worrying …”. Some other nurses reported feelings of helplessness. They felt that “… nothing can be done to improve current situation…” and that they have “… no interest in doing activities …”.

#### 3.1.4. Stress-Related Coping Strategies

The fourth category was stress-related coping strategies and consisted of four codes: (1) medication, (2) talking to others, (3) relaxation and (4) suppression of thoughts. Most of the ICU nurses who participated in the study reported that they talked to others about the pandemic experience to lighten the load of the concerns they had. Participants statements indicated the importance of seeking support from families, colleagues and friends in empowering them and help in improving their ability to adapt to stress. Nurses mainly talked with either “… family member …” or “… close friend …”. Some nurses sought medical advice to get “… prescribed pills …” or just buy “…over the counter supplement …” to help them sleep and relax. Most of the ICU nurses reported that the best way to relax or de-stress is reading or listening to books, particularly spiritual books. Participating nurses also used the technique of “… clearing the thoughts …” in which they consciously attempt to “… stop thinking …” about distressing thoughts.

## 4. Discussion

Healthcare organizations worldwide, during the COVID-19 pandemic, have faced extreme, often unavoidable, challenges. Similar to other countries, the Ministry of Health (MOH) in the Kingdom of Saudi Arabia (KSA) at the beginning of the first wave of the COVID-19 pandemic had declared some of its hospitals to be completely “COVID-19” hospitals. These hospitals were rapidly transformed into isolation hospitals with critical care capacity. This decision has indeed offered much of the needed spare capacity during the first and second waves of the COVID-19 outbreak. In these hospitals, the work environment of ICU nurses during the pandemic included close contact with patients who were infected with the virus and strict protection. The physical and psychological work risks they faced were characterized as high. The KSA government attached great importance to protecting ICU nurses from infection as well as maintain their physical and mental well-being. However, there were still staff members who were unfortunately presented with compromised physical health and psychological trauma experiences [26]. 

This is a secondary analysis of existing data, obtained from a larger quantitative study conducted after the end of the 1st wave of COVID-19 to identify the prevalence of posttraumatic stress disorder (PTSD) among ICU nurses who were assigned to COVID-19 hospitals. Reflecting on the quantitative results, PTSD is common among the ICU nurses who participated in the study. PTSD can negatively impact the quality of life [27]. Indeed, the literature showed that around 5% of nurses are depressed and 15% are anxious [28]. However, frequent screening for PTSD showed the likelihood of spontaneous remission. Indeed, a study conducted during the peak of COVID-19 pandemic by Liu et al. [10] showed that the prevalence of PTSD symptoms among healthcare workers was 7%. A few months later, the Liu et al. (2020) study was followed by a study conducted by Yin et al. [29] to assess the same healthcare workers, and that study shows that the prevalence of PTSD symptoms has dropped to 3.8%.

The secondary analysis of existing data aimed to explore the experiences of ICU nurses after providing direct care for COVID-19 patients. Due to the critical nature of the pandemic, seeking an in-depth understanding of the phenomenon without the limits of traditional quantitative measures using qualitative manifest content analysis is highly recommended [26].

ICU nurses have a higher risk of getting infected with the virus while caring for patients with COVID-19 than nurses working in other units. Thus, they have chosen to live separately to reduce the risk of affecting their family and other people in their close surroundings [10]. In the current study, participating ICU nurses reported experiencing stressful times while working in COVID-19 hospitals. Specifically, they reported that being unable to maintain physical contact with their family members was the hardest part of the pandemic experience. 

Many of ICU nurses on the front lines of COVID-19 slept in furnished apartments and hotel rooms for months instead of returning to their homes [30]. Others had no choice but to isolate at home after going through careful self-decontamination procedures [31]. Due to self-isolation from loved ones, health professionals lacked the essential psychological support that normally helps people to manage stressful personal, work and life issues. This may result in a high risk for PTSD for ICU nurses on the front lines of COVID-19 [32].

Several studies on healthcare professionals worldwide confirm the results of the current study concerning that family support is essential in maintaining the psychological well-being of individuals during COVID-19 lockdown [33,34].

Given that the majority of participants in the current study were women and married, it was not surprising that the statement “it was hard staying away from kids” was the most frequently repeated. This may show the intensity of the interruption of the parent/child relationship [35]. Further, under normal circumstances, separation is considered one of the critical causes for disruption in the relationship between partners. The fact that partners have fights over small things after the pandemic, as reported by some participants in this study, may raise critical concerns. This might be a sign of failure to cope with the crisis [35]. Perhaps giving healthcare professionals the chance to take breaks from work and reconnect with their partner would provide them the opportunity to meet their physical, mental and emotional needs [35].

For health professionals, the death of a colleague can be particularly traumatic, and when this loss is combined with long working hours, reduced time for rest and a high rate of death in the working environment, huge emotional and interpersonal stressors can fall upon the shoulders of ICU nurses [36]. The literature has shown that working under such circumstances may worsen preexisting burnout responses [37].

Constant stressful events are harmful to nurses’ physical, emotional and mental health [38]. As a consequence, an increased number of nurses are thinking about changing their working area or even leaving the profession [39]. In the current study, this was supported by the quantitative results as approximately 54% of the total ICU nurses who participated in the survey were thinking of leaving the job. The prevention of work stress, which is becoming unavoidable for those working with COVID-19 patients, is a major challenge mainly for the health institution. Reducing the experience of stress and improving psychosocial working conditions may contribute to maintaining ICU nurses’ mental health and wellbeing, as well as maintaining their ability to work effectively [40].

Helping front-line ICU nurses to adjust to traumatic events during work is highly recommended [41]. Studies showed that after a period of constant stress, individuals gradually adapted to their status [42]. In this regard, the World Health Organization [43] issued recommendations to enhance and accelerate the resilience process of clinicians on the front lines, including but not limited to maintaining good quality of communication, rotating healthcare workers from higher-stress areas to lower-stress areas and pairing inexperienced healthcare workers with their more experienced colleagues.

The participants in the study reported several traumatic distress signs: sleep disturbance and insomnia, distress, social isolation, fear and helplessness. This is not surprising, since ICU nurses during COVID-19 managed a greater number of patient deaths than usual and experienced fear of the disease and uncertainty of the outcomes of the pandemic [44]. The study findings are aligned with the results of many studies reported during and shortly after the COVID-19 pandemic [27]. In a comparative study, Ceri and Cicek [45] claimed that nurses exposed to patients with COVID-19 were more vulnerable to some interacting psychological symptoms such as sleep disturbance and depression. These symptoms may sometimes be followed by the intention to leave the job [46]. Infecting their family members, friends and colleagues with COVID-19 virus was found to be a major source of stress for healthcare workers [47].

The participants in the study reported that they used several coping techniques to reduce stress caused by the pandemic: taking sleeping pills to help them sleep, talking about their stress and sharing their negative feelings with others, using religious beliefs or practices such as reading holy books, trying to create own calming space and avoiding negative thoughts. These coping strategies are to some degree similar to those used during other pandemic diseases (e.g., the Ebola pandemic) [48]. Coping strategies add to the psychological health of people affected by disasters [49]. This implies the importance of considering training on coping skills [50].

The findings of this study will add knowledge to the small body of the literature on the experiences of ICU nurses during the COVID-19 pandemic. The qualitative evidence of the lived experience of ICU nurses can illustrate how the circumstances of these nurses from their perspective may compound or mitigate the risk of mental health problems caused by the pandemic. It is hoped that the findings provide a new theoretical basis for psychological intervention for ICU nurses who may experience a future traumatic surge.

### 4.1. Strengths and Limitations

The main strength of the study is its rigor. Indeed, although there are many published qualitative studies which have explored the same area of investigation, the current study used exploratory explicit qualitative content analysis. This analysis technique meets the scientific quality criteria by demonstrating a low abstraction and a low interpretation level. Indeed, a lower level of abstraction leads to lesser distance from the original text. This approach also has high reliability.

The researchers would like to acknowledge the limitation that this is secondary analysis of existing data, and no statistical relationship was sought between the findings and the collected demographic data. Although the current study provides an important insight into the COVID-19 experiences of ICU nurses by including several open-ended questions with the quantitative survey, the study researchers wished to limit the number of open-ended questions. Such a limitation of the number of questions may have influenced the number of issues being raised or explored. It is believed that saturation was achieved and that additional open-ended questions would not reveal new categories. Yet, there is a need to conduct a mixed-method study design in order to establish relationships emerging from the quantitative data of participant characteristics and the prevalence of PTSD among ICU nurses and the qualitative data of stressful factors and management strategies.

The current study did not investigate the relationship between nurses working in other wards and those brought for the first time to the ICU during the pandemic and their stress level. This could add additional insight if investigated. Finally, this study was conducted in a limited geographic region; therefore, the findings may not be applicable in all sociodemographic strata and cultures, and therefore they cannot be generalized.

### 4.2. Implications

The findings from this study can be translated into several practical recommendations. First, implementing continuous training and providing psychological guidance for ICU nurses can contribute to protecting them from collapsing due to unmanageable emotions and stress. An example of continuous training could be mindfulness meditation practice and stressful situational simulation exercises. Simulated exercises of stressful events may help to train ICU nurses to better deal with emergency surges. Second, enhancing communication between nurses and their families during emergency traumatic events, can help avoid the adverse effects on their well-being, especially with regard to peers and close family members. Such enhancement may have a significant impact on nurses’ mental health. Alternatively, electronic applications are very important when physical visits are not possible. Switching front-line nurses with nurses working in other areas, during stressful emergency surges, provides the ICU staff the opportunity to rest, re-connect with family and become emotionally re-energized. Finally, the setup of support agencies, specifically for the mental well-being of healthcare professionals, may facilitate developing and implementing mental health intervention strategies.

## 5. Conclusions

This study provides important insights into the experience of ICU nurses during the COVID pandemic. This can offer valuable lessons for nurses and decision-makers. One of the lessons is to give nurses’ psychological well-being adequate attention from hospital decision-makers during stressful emergency events. Additionally, the other significant lesson has been recognizing the important role of ICU nurses’ family support and religious–spiritual practices during the pandemic. There is, therefore, a need for family-friendly policies and interventions to be taken into consideration to use the strengths of the intrafamilial process for coping in times of future pandemics. Further research is needed to understand the long-term effects of COVID-19-related stressful events and to maintain the well-being of ICU nurses during stressful emergency surges in intensive care.

## Figures and Tables

**Table 1:** Manifest content analysis.

Word(s)/Phrase(s)	Freq. Count	Codes	Category
Stay away from … kid(s)	10 time(s)	Family separation	Family-related stressful factors
Living apart from … husband	3 time(s)		
Not able to be near … parent(s)	5 time(s)		
Stop seeing … sister(s)/brother(s)	2 time(s)		
Not be able to be close to … grandmother(s)	1 time(s)		
Heavy smoker … husband	1 time(s)	Fear of transmitting infection to vulnerable family members	
Thyroid … sister(s)	1 time(s)		
Diabetic … parent(s)	3 time(s)		
Old … grandmother (s)	1 time(s)		
Mother … death	1 time(s)	Death of a loved one	
Fighting with husband	1 time(s)	Marital problems	
Caring for more critical patients at the same time	10 time(s)	Workload	Work-related stressful factors.
Less rest time … days off	11 time(s)		
Death from COVID-19	2 time(s)	Death of patient	
Increase deaths (s)	1 time(s)		
Physician(s) death	1 time(s)	Death of a colleague	
Difficult to sleep	7 time(s)	Sleeping difficulties	Stress-related psychosocial signs
Sad	3 time(s)	Distress	
Hard time	10 time(s)		
Separating self from others	1 time(s)	Social-isolation	
Avoid joining family, friends’ occasions	2 time(s)		
Constant worrying	1 time(s)	Fear	
Nothing can be done	1 time(s)	Helplessness	
No interest in doing activities	1 time(s)		
Prescribed pills	1 time(s)	Medication	Stress-related coping strategies
Over the counter supplement	6 time(s)		
Speak to family member	16 time(s)	Talk to other	
Chat with friend	8 time(s)		
Reading/listening to holy book(s)	13 time(s)	Relaxation	
Clearing thoughts	1 time(s)		
Stop thinking	1 time(s)	Suppress the thoughts	

## Data Availability

All data generated or analyzed during this study are included in this published article.
